# Acidum Benzoicum

**Published:** 1885-01

**Authors:** 


### ACIDUM BENZOICUM.

General Characteristics.—These are generally found in the urine, which is scanty, of a dark brown color, and the *urinous odor being highly intensified.* In relation to the strong smell of the urine, care must be taken not to fall into error by a strong smell emanating from urine which has been kept covered for a long time (or over night), or which has remained on sheets which have not been changed recently. The urine must have this characteristic smell when *freshly voided.* The color of the urine is not of so much importance as the smell; for the color may be different.

Nocturnal eneuresis, with the above characteristic odor, sheets usually stained brown.

Rheumatism, quinsy, dropsy, diarrhœa, headache, all when accompanied by this *highly intensified odor of the urine.* Menstrual difficulties, when accompanied by the characteristic smell of the urine.

Sides.—Most of the symptoms appear on the left side, but may frequently come on the right side.

Aggravations.—Headache worse when at rest; toothache when lying down; eye and ear symptoms worse in open air; on uncovering head.

Amelioration.—From heat.—G.

